# Trehalose and Dry Eye Disease: A Comprehensive Systematic Review of Randomized Controlled Trials

**DOI:** 10.3390/jcm12237301

**Published:** 2023-11-25

**Authors:** Antonio Ballesteros-Sánchez, Clara Martinez-Perez, Cristina Alvarez-Peregrina, Miguel Ángel Sánchez-Tena, Concepción De-Hita-Cantalejo, María Carmen Sánchez-González, José-María Sánchez-González

**Affiliations:** 1 Department of Physics of Condensed Matter, Optics Area, University of Seville, 41004 Seville, Spainmsanchez77@us.es (M.C.S.-G.); 2 Department of Ophthalmology, Clínica Novovisión, 30008 Murcia, Spain; 3 ISEC LISBOA (Instituto Superior de Educação e Ciências), 1750-142 Lisbon, Portugal; clara.perez@iseclisboa.pt; 4 Optometry and Vision Department, Faculty of Optics and Optometry, Complutense University of Madrid, 28037 Madrid, Spain

**Keywords:** trehalose, hyaluronic acid, tear substitutes, dry eye disease

## Abstract

The purpose of the research project was to extensively review the efficacy and safety of a trehalose tear-substitute treatment in cases of dry eye disease (DED). A systematic review that included only full-length randomized controlled studies (RCTs) reporting the effects of trehalose tear-substitute treatment in three databases, PubMed, Scopus and Web of Science, was performed according to the PRISMA statement. The search period included papers published before 8 August 2023. The Cochrane risk-of-bias tool was used to analyze the quality of the studies selected. A total of 10 RCTs were included in this systematic review. Trehalose tear-substitute treatments achieved a higher improvement than did control group interventions in all reported variables. The mean differences between both groups were in favor of trehalose, and were as follows: ocular surface disease index (OSDI) questionnaire score of −8.5 ± 7 points, tear film breakup time (TBUT) of 1.9 ± 1 s, tear film thickness (TFT) of 0.25 ± 0.1 μm, tear meniscus height (TMH) of 0.02 ± 0.02 mm, Schirmer test (ST) of 0.8 ± 1.4 mm, corneal fluorescein staining (CFS) of −0.7 ± 0.1 points and visual acuity (VA) of 0.3 ± 2.1 letters. No adverse events after trehalose tear-substitute treatments were reported. Trehalose tear substitutes are a safe and effective treatment for DED. Therefore, trehalose tear substitutes should be recommended for patients with dry eye disease. In addition, there is specific evidence to support its use in the preoperative cataract surgery period.

## 1. Introduction

Dry eye disease (DED) is a chronic, multifactorial and inflammatory disease of the ocular surface that affects up to 30% of adults over the age of 50 and is more frequent in women; additionally, its prevalence increases with age [1,2]. DED is characterized by a reduced production or excessive evaporation of tears, which leads to symptoms such as dryness, irritation, burning, and blurred vision [3]. DED can have a significant impact on the quality of life of affected individuals and is a leading cause of visits to eye care professionals worldwide [4,5].

The management of DED involves a range of treatments aimed at relieving symptoms, improving tear film stability, and reducing inflammation [6,7]. According to the management and therapy subcommittee of the Tear Film and Ocular Surface (TFOS) Dry Eye Workshop (DEWS) II, tear substitutes constitute the first-step therapy for DED [6]. However, this treatment has limitations, and many patients continue to experience symptoms despite treatment [6,8]. Therefore, new tear-substitute formulations are under research. Trehalose, a natural disaccharide consisting of two glucose molecules, has been shown to have protective properties against various stressors and is known to stabilize proteins and membranes, prevent denaturation and inhibit oxidative damage [9]. Trehalose has been found to have several potential benefits for the management of DED [10]. Firstly, trehalose can stabilize the lipid layer of the tear film, which is essential for maintaining tear-film stability and preventing evaporation [11,12]. Secondly, trehalose can reduce tear-film osmolarity, which is often elevated in DED and contributes to ocular surface damage [13]. Thirdly, trehalose has been shown to enhance the survival of corneal epithelial cells, which are essential for maintaining the integrity and function of the ocular surface [13,14]. Lastly, trehalose has anti-inflammatory properties and can modulate the expression of genes involved in the regulation of tear secretion and ocular surface homeostasis [14,15].

To date, some published studies have evaluated the effects of trehalose tear substitutes in DED. However, to the best of our knowledge, no systematic reviews have explored the available literature regarding the benefits of trehalose tear-substitute treatments. Therefore, the objective of this systematic review is to evaluate the efficacy and safety of trehalose tear substitutes in the management of DED based on the available randomized controlled trials (RCTs). Through this review, we aim to provide a comprehensive overview of the current evidence on trehalose, enabling evidence-based decision making and guiding future research directions.

## 2. Materials and Methods

### 2.1. Data Sources and Search Strategy

This systematic review was performed according to the preferred reporting items for systematic reviews and meta-analyses (PRISMA) method [16,17]. We identified 136 articles published before 8 August 2023 through the following databases: PubMed, Scopus and Web of Science. The data search strategy with Boolean operators was as follows: (trehalose OR trehalose solution) AND (artificial tears OR tear substitutes OR eye drops) AND (dry eye disease OR dry eye syndrome OR DED OR dysfunctional tear syndrome OR keratoconjunctivitis sicca). These keywords were selected by consensus among the researchers. The references of the retrieved articles were reviewed to identify other related studies that might meet the inclusion criteria.

### 2.2. Study Selection

All of the 136 articles identified through the search strategy were considered and analyzed. Duplicate studies were removed by DistillerSR software (Version 2.35, DistillerSR Inc., Ottawa, ON, Canada) [18]. The remaining studies underwent additional screening stages, which included title screening, abstract screening and full-text screening. Studies unrelated to the topic were excluded from the review during title and abstract screenings. In full-text screening, studies that did not include the instillation of trehalose eye drops were also excluded from the review. The studies were reviewed by two researchers (ABS and JMSG), who selected them according to the inclusion and exclusion criteria. In cases of discrepancies between the reviewers, a third author was consulted to resolve the disagreement.

The inclusion criteria were as follows: human studies, full-length original articles, and RCTs. The exclusion criteria excluded non-English-language publications and unindexed journals. There were no restrictions set as to the country in which the study was performed, the follow-up period, the sample size, or the results of the study.

### 2.3. Quality Assessment and Data Extraction

The data from each study were collected and summarized independently in tables designed by two researchers (ABS and JMSG). The following information was obtained from each article: (1) author and date of publication (year), (2) study design, (3) mean follow-up duration for all patients in the whole procedure (expressed in hours or months), (4) number of patients, (5) mean age of the patients (expressed in years), (6) patient sex (male/female), (7) number of eyes involved, (8) study group intervention, (9) control group intervention, (10) adverse events and (11) conflicts of interest.

Regarding the results of the studies, the following data were collected according to TFOS DEWS II for DED diagnosis [19]: (14) ocular surface disease index [(OSDI), values from 0 to 100] [20]; (15) tear break-up time [(TBUT), expressed in seconds (s)]; (16) tear film thickness [(TFT), expressed in microns (μm)]; (17) tear meniscus height [(TMH), expressed in millimeters (mm)]; (18) Schirmer I test [(ST), expressed in millimeters (mm)] [19]; (19) corneal fluorescein staining [(CFS), assessed with Oxford grading score] [21]; (20) patient satisfaction (defined as “no satisfaction”, “low satisfaction”, “moderate satisfaction” and “high satisfaction”); and finally, (23) the authors’ judgment as expressed by commenting in favor or against the use of trehalose eye-drops treatments. Baseline and last-visit values for all these variables were collected in the treatment (T) and control (C) groups. Intra-group clinical outcomes were defined as “Last visit (LV)—Baseline (B) differences”. Inter-group clinical outcomes were defined as “T group _(LV–B)_—C group _(LV–B)_ differences”. Mean ± SD, [range] for each variable were calculated to report intra-group and inter-group clinical outcomes.

The literature that remained after full-text screening was examined to assess the quality of the studies. To avoid the risk of bias, two dependable authors created a synopsis based on the Cochrane risk-of-bias tool [22] which included the following items: (1) random sequence generation, (2) allocation concealment, (3) blinding of participants and personnel, (4) blinding of outcome assessment, (5) incomplete outcome data, (6) selective reporting and (7) other sources of bias. A third nonblinded assessor decided the quality of the studies when disagreements occurred between the two assessors. This assessment did not determine the exclusion of any study.

## 3. Results

### 3.1. Study Characteristics

**Figure 1:**
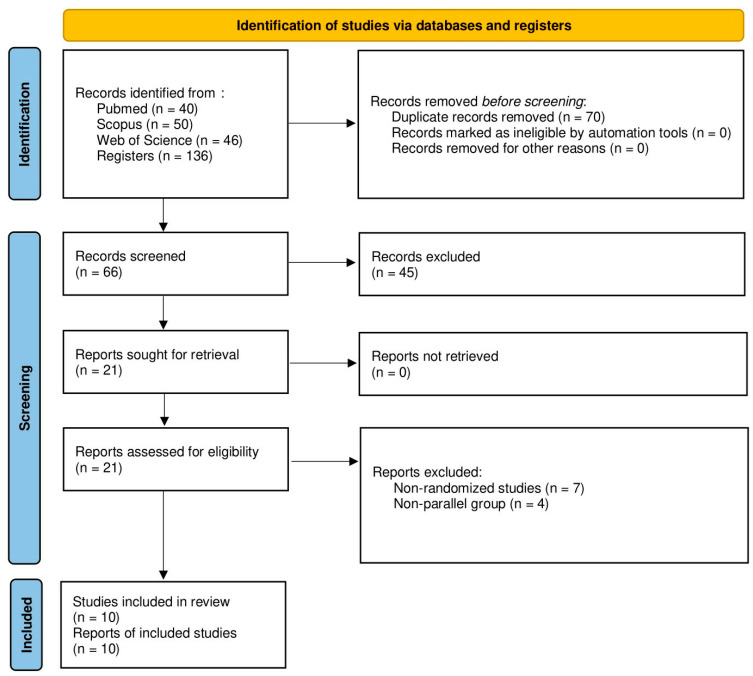
Flowchart study selection process according to the PRISMA statement.

The study-selection process of this systematic review is presented with a flowchart diagram in Figure 1. The design of the included studies comprised prospective randomized controlled trials published between 2015 and 2021. This systematic review included 1252 patients with a mean age of 56.9 ± 10.8 years. The sex distribution was 787 females (66.1%) and 405 males (33.9%). One study did not report age and sex distribution [23]. Patient follow-up, expressed in months, ranged from 1 month [23,24] to 8 months [15], with a mean follow-up of 2.7 ± 2.3 months. Regarding study group intervention, six studies used Thealoz duo^®^ (Laboratories Théa, Ferrand, France) [12,13,15,24,25,26], three studies used Thealoz duo gel^®^ (Laboratories Théa, Ferrand, France) [11,23,27] and one study used Trehalube^®^ (Micro Labs, Bangalore, India) [28]. Different interventions were used in the control group, such as Hydrabak^®^ (Laboratories Théa, Ferrand, France) [12,13,23], Hyabak^®^ (Laboratories Théa, Ferrand, France) [12,15], Hylo gel^®^ (Brill Pharma, Barcelona, Spain) [11], Hylotears^®^ (Ursapharm, Sarrebruck, Germany) [28], Systane gel drops^®^ (Alcon, Barcelona, Spain) [11], Vismed^®^ (Brudy Lab, Barcelona, Spain) [25,26], Vismed gel^®^ (Brudy Lab, Barcelona, Spain) [27], and no intervention [15,24]. Three studies had conflicts of interest (supported by Laboratories Théa, Ferrand, France) [11,25,26]. More detailed study characteristics and tear-substitute compositions are presented in Table 1.

### 3.2. Outcomes 

Regarding outcomes measures, six studies reported dry-eye symptom outcomes [13,15,23,24,25,26]. Also, nine studies reported dry-eye sign outcomes [11,12,13,15,23,24,25,27,28], of which seven studies evaluated TBUT [12,13,15,23,24,25,28], six studies assessed ST [12,13,15,24,25,28], four studies evaluated CFS [13,15,23,25], three studies measured VA [12,15,23] and two studies assessed TFT [11,12] and TMH [27,28]. Patient satisfaction was reported by four studies [11,23,25,27].

Intra-group clinical outcomes are presented in Table 2 and Table 3. Regarding treatment groups, most of the outcomes achieved an improvement, with a mean OSDI questionnaire score of −22.4 ± 12.2, [−36.7 to −4.6] points, mean TBUT of 2.8 ± 1.2, [0.9 to 4.2] s, mean TFT 0.5 ± 0.2, [0.3 to 0.7] μm, mean ST of 2.8 ± 1.2, [1.6 to 5.3] mm, mean CFS of −1.1 ± 0.2, [−1.2 to −0.8] points and mean VA of 10.6 ± 8.9, [−2.1 to 17.6] letters. TMH remained almost unchanged, with a mean value of 0.08 ± 0.1, [0.06 to 0.1] mm. Regarding the control group, most of the outcomes also achieved an improvement, with a mean OSDI questionnaire score of −14.3 ± 10.1, [−28.2 to 1.2] points, mean TBUT of 0.9 ± 1, [−0.3 to 2.6] s, mean TFT of 0.25 ± 0.2, [0.1 to 0.4] μm, mean ST of 1.9 ± 1.6, [−0.5 to 3.7] mm, mean CFS of −0.4 ± 0.2, [−0.8 to −0.2] points and mean VA 10.2 ± 7, [0.4 to 16.3] letters. TMH also remained almost unchanged, with a mean value of 0.05 ± 0.1, [−0.02 to 0.1] mm.

Inter-group clinical outcomes are presented in Table 4. All outcomes were in favor of the treatment group, with a mean OSDI questionnaire score of −8.5 ± 7, [−20 to −1.7] points, mean TBUT of 1.9 ± 1, [0.1 to 4.5] s, mean TFT 0.25 ± 0.1, [0.2 to 0.3] μm, mean ST of 0.8 ± 1.4, [−1.1 to 3.3] mm, mean CFS of −0.7 ± 0.1, [−0.8 to −0.6] points, mean VA of 0.3 ± 2.1, [−2.5 to 2.3] letters and mean TMH of 0.02 ± 0.02, [0.00 to 0.02] mm. Regarding adverse events, six studies reported no adverse events after trehalose tear-substitute treatments [11,12,13,15,28]. In addition, four studies reported high patient satisfaction after trehalose tear-substitute treatments.

### 3.3. Risk of Bias 

**Figure 2:**
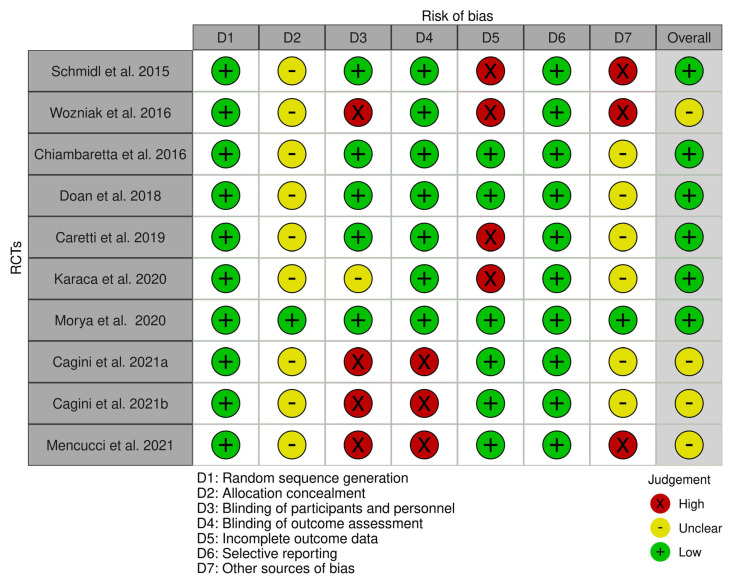
Risk-of-bias summary for the included studies, with traffic-light plot. The traffic lights represent the authors’ risk of biased judgment in each domain (D) used to assess the quality of the studies [11,12,13,15,23,24,25,26,27,28].

**Figure 3:**
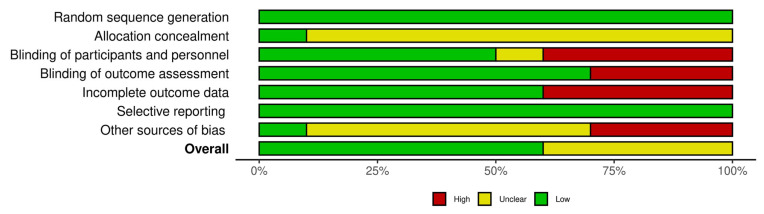
Overall risk-of-bias summary of the domains, with bar plot. Bars represent the authors’ overall risk of biased judgment in each domain, presented as percentages.

The risk-of-bias summary of the included studies is presented in Figure 2. Risk-of-bias assessment was classified into three evidence-level groups: (1) studies with a low risk of bias (Schmidl et al. [12], Chiambaretta et al. [25], Doan et al. [26], Caretti et al. [23], Karaca et al. [27] and Morya et al. [28]), (2) studies with an unclear risk of bias (Wozniak et al. [11], Cagini et al. [13,15] and Mencucci et al. [24]) and (3) studies with a high risk of bias (no studies). The overall risk-of-bias summary of the domains used in each study is presented in Figure 3. The items used to assess the risk of bias showed an overall low risk of bias for over 50% of the included analyses. The Robvis tool (NIHR, Bristol, UK) was used to create risk-of-bias assessment figures [29].

## 4. Discussion

Tear film instability is considered the trigger for the ocular surface inflammatory mechanisms that lead to the signs and symptoms of dry eye [3]. Tear substitutes are usually the first line of treatment for patients with DED [6]. Therefore, new formulations that improve the tear film quality and restore the homeostasis of the ocular surface are the subjects of constant research [30]. This systematic review aimed to report the effects of trehalose tear-substitute treatments on the signs and symptoms of dry eye.

### 4.1. Trehalose Efficacy in DED

Although there are different questionnaires to assess dry-eye symptoms, the OSDI questionnaire is the most widely used for DED studies, and it is validated in different languages. Chiambaretta et al. [25] and Doan et al. [26] reported significant OSDI score improvement in both groups, but the results were in favor of the trehalose group, with differences of −4.6 and −1.7 points, respectively. The control groups received hyaluronic acid (HA) 0.18% tear substitutes, which may explain the OSDI score improvements. HA is a linear polysaccharide which has been shown to be superior in increasing tear film viscosity and stability compared to other tear-substitute formulations, resulting in greater relief of dry-eye symptoms [31,32]. However, Doan et al. [26] reported that 78.8% of patients in the trehalose group achieved an OSDI score below 19 points, compared to 58.5% in the HA group, after 3-month follow-up.

Tear film stability and volume, as well as damage to the ocular surface, are tests recommended by the TFOS DEWS II for DED diagnosis [19]. Regarding tear film stability, Schmidl et al. [12], Chiambaretta et al. [25] and Morya et al. [28] reported that the trehalose groups achieved TBUT improvements of 0.4 s, 0.1 s and 1.1 s compared to the control groups, respectively. Morya et al. [28] was the only study that reported significant TBUT improvements in the trehalose group, which may be explained by the large sample size.

Regarding tear-film volume, Schmidl et al. [12] and Wozniak et al. [11] showed that the trehalose group achieved higher TFT measurements, of 0.3 μm and 0.2 μm, respectively, compared to those of the control groups. In addition, Schmidl et al. [12] and Wozniak et al. [11] also reported that HA 0.15% and propylene glycol (PG) 0.4% only increased TFT for 35 ± 5 min, while trehalose increased TFT for as long as 180 ± 60 min. This may be explained as being due to the interaction of trehalose with lipid membranes, which involves the surrounding hydration shell, where the unique disaccharide structure of the trehalose stabilizes the membrane by replacing water molecules and enhancing the membrane’s resilience to stress, thereby maintaining the membrane’s integrity and functionality [33]. Karaca et al. [27] reported a significant TMH improvements, of 0.1 mm, in the trehalose group. The control group also achieved significant TMH improvements because they received HA 0.3%, which may match the effect of trehalose due to its spreading, pseudoplasticity and muco-adhesion properties [34,35,36]. Karaca et al. [27] also reported that trehalose and HA 0.3% increased TMH for 90 ± 30 min. However, Morya et al. [28] showed that TMH remained unchanged in both groups after two-month follow-ups. This suggests that tear substitutes’ effects on TFT and TMH are only sustained for a few hours after instillation and not for long time periods. Morya et al. [28] reported that the trehalose group achieved an ST improvement of 1.6 mm compared to the control group, while Schmidl et al. [12] and Chiambaretta et al. [25] reported a ST improvements of 1.1 mm and 0.7 mm, respectively, in favor of the control groups. It is important to mention that these studies performed the ST without anesthesia; therefore, the results are not reliable, due to the action of reflex tearing [19]. In addition, Li et al. [37] reported that ST after topical anesthesia was significantly lower than ST without anesthesia, concluding that ST with topical anesthesia is more objective and reliable for the diagnosis and treatment of DED.

Damage to the ocular surface was assessed by the CFS. Chiambaretta et al. [25] was the only study that reported CFS changes in patients with DED after tear-substitute instillation. They reported significant CFS reduction in both groups, but the results were in favor of trehalose group, with a difference of −0.6 points [25]. Trehalose is synthesized during prolonged periods of desiccation to protect cells from dehydration [38]; this dehydration seems to occur in corneal epithelial cells [39,40]. This osmoprotective effect of trehalose may help to reduce the corneal epithelial staining and restore the ocular surface [41].

### 4.2. Trehalose Efficacy in Cataract Surgery

Cataract surgery with intraocular lens implantation is a safe and effective procedure that improves visual acuity, with very high patient satisfaction [42,43]. Caretti et al. [23] and Cagini et al. (2021b) [15] reported significant VA improvements in both groups, of 16.9 ± 0.6 letters and 15.2 ± 1.2 letters, respectively. However, it is well known that several complications can develop after surgery, such as dry eye [44,45]. The surgical procedure may have an impact on the tear film and the ocular surface [46], inducing or exacerbating dry-eye symptoms which significantly affect patients’ quality of life [47,48]. In addition, Miura et al. [49] reported that more than one-third of patients without preexisting DED developed DED after cataract surgery. Therefore, prevention and treatment of cataract-surgery-induced dry eye should be considered [44,45,47,48,50].

Caretti et al. [23], Cagini et al. (2021a) [13] and Cagini et al. (2021b) [15] evaluated the effects of trehalose tear substitutes on tear film stability and volume, as well as on ocular surface damage after cataract surgery. Caretti et al. [23], Cagini et al. (2021a) [13] and Cagini et al. (2021b) [15] reported OSDI, TBUT and CFS improvement in both groups, but the results were in favor of the trehalose group, with differences of −16.3 points, −2.5 points and −20 points for OSDI; 2.2 s, 4.5 s and 3.3 s for TBUT; and −0.6 points, −0.6 points and −0.8 points for CFS, respectively. Cagini et al. (2021a) [13] and Cagini et al. (2021b) [15] also showed ST improvements in both groups. However, neither study specifies whether the ST was performed with anesthesia, and thus the results may not be reliable, due to the action of reflex tearing. In addition, Caretti et al. [23] and Cagini et al. (2021b) [15] reported that patients who received postoperative treatment of trehalose tear substitutes achieved VA improvements of 1.2 letters and 2.3 letters, respectively, compared to those who received either HA 0.15%, sodium chloride (NaCl) 0.9%, or no tear substitutes. Mencucci et al. [24] was the only study that evaluated the effects of trehalose tear-substitute treatments before cataract surgery. They reported that patients who received preoperative and postoperative treatments of trehalose tear substitutes achieved significant OSDI score and TBUT improvements of −5.8 points and 1.5 s, respectively, compared to those who only received postoperative treatments of trehalose tear substitutes or no tear substitutes. Mencucci et al. [24] also assessed ST without anesthesia, which may influence the results, due to the action of reflex tearing [19].

### 4.3. Trehalose Safety

Schmidl et al. [12], Doan et al. [26], Morya et al. [28], Cagini et al. (2021a) [13], Cagini et al. (2021b) [15] and Mencucci et al. [24] did not report patient satisfaction after trehalose tear-substitute treatments. Wozniak et al. [11], Chiambaretta [25], Caretti et al. [23] and Karaca et al. [27] reported that all patients who received trehalose tear substitutes experienced a high level of overall satisfaction. Although trehalose is a naturally bioactive sugar [9], mammalian cells cannot synthesize it [51]. However, the non-toxicity of trehalose allows its administration in humans, with minimal adverse effects [38,51]. In addition, no adverse events after trehalose tear-substitute treatments were reported by the articles included in this systematic review.

### 4.4. Strengths and Limitations

The main strength of this systematic review is in the results obtained, due to the fact that all studies included were RCTs with a low overall risk of bias. The main limitation of our review is the heterogeneity of the interventions in both groups, including the treatment combinations of trehalose with hyaluronic acid or other components, which complicated comparisons between the included studies. In addition, the dose applied per day differs among the studies; thus, the methodologies of all the studies were not remarkably similar. The short follow-up period is also a limitation that may have influenced the results reported by the studies included. Therefore, larger, well-designed, strictly blinded, multicenter RCTs with extensive follow-up are needed to determine the safety and efficacy of trehalose tear-substitute treatments alone versus trehalose tear-substitute treatments combined with other osmoprotectants, such as glycine or betaine, and their respective effective durations over time.

## 5. Conclusions

In conclusion, this systematic review has demonstrated that trehalose tear-substitute treatments achieve better results than the traditional tear substitutes, reporting high patient satisfaction with no adverse events. Therefore, trehalose tear substitutes are an effective and safe treatment that should be recommended for DED. Trehalose tear substitutes improve DED symptoms and signs such as OSDI score, TBUT, TFT and CFS. However, there is insufficient evidence to suggest that trehalose tear substitutes improve TMH and ST. Regarding trehalose tear-substitute treatments in cataract surgery, it seems to be more beneficial to use trehalose tear substitutes during the preoperative and postoperative period. Therefore, this treatment should be considered in cataract surgery, especially in patients with preexisting DED.

## Figures and Tables

**Table 1:** Summary of included RCTs.

Author, Date	Design	F/U	Patients (TG/CG)	Age ^c^ (TG/CG)	Sex (F/M)	Eyes	Inclusion Criteria	Intervention	Control	Adverse Events	CoI
Schmidl et al. [12] 2015	MN SM	4 ^a^	60 (20/40)	42.7 ± 11.6 (43.6 ± 13.3/42.4 ± 11)	43/17	60	DED	Thealoz Duo (TH 3% and HA 0.15%)	Hyabak (HA 0.15%) or Hydrabak (NaCL 0.9%)	No	No
Wozniak et al. [11] 2016	MN SM	6 ^a^	60 (20/40)	45.6 ± 13.5 (45 ± 13.1/45.9 ± 13.8)	37/23	60	DED	Thealoz Duo gel (TH 3%, HA 0.15% and CB 0.25%)	Hylo gel (HA 0.2%) orSystane gel (PG 0.4%)	No	Yes
Chiambaretta et al. [25] 2016	MT SM	3 ^b^	105 (52/53)	59.2 ± 12.8 (60 ± 12.2/58.5 ± 13.4)	86/19	105	DED	Thealoz Duo (TH 3% and HA 0.15%)	Vismed (HA 0.18%)	No	Yes
Doan et al. [26] 2018	MT SM	3 ^b^	105 (52/53)	59.2 ± 12.8 (60 ± 12.2/58.5 ± 13.4)	86/19	105	DED	Thealoz Duo (TH 3% and HA 0.15%)	Vismed (HA 0.18%)	NR	Yes
Caretti et al. [23] 2019	MN DM	1 ^b^	60 (30/30)	NR	NR	60	DED Cataract	Thealoz Duo gel (TH 3%, HA 0.15% and CB 0.25%)	Hydrabak (NaCL 0.9%)	NR	No
Karaca et al. [27] 2020	MN SM	4 ^a^	122 (56/66)	52.9 ± 12.3 (52.5 ± 13.2/53.4 ± 11.4)	104/18	122	DED	Thealoz Duo gel (TH 3%, HA 0.15% and CB 0.25%)	Vismed gel (HA 0.3%)	NR	No
Morya et al. [28] 2020	MN SM	2 ^b^	384 (192/192)	37.6 ± 14.4 (36.7 ± 14.8/36.1 ± 13.6)	224/160	384	DED	Trehalube (TH 3%, HA 0.1%)	Hylotears (HA 0.1%)	No	No
Cagini et al. [13] 2021a	MN UM	1 ^b^	135 (66/69)	73.7 ± 4.2 (73.2 ± 4.5/74.3 ± 3.8)	82/53	135	DED Cataract	Thealoz Duo (TH 3% and HA 0.15%)	Hydrabak (NaCL 0.9%)	No	No
Cagini et al. [15] 2021b	MN UM	8 ^b^	98 (33/65)	67.7 ± 6.9 (65.4 ± 7.2/64.8 ± 5.8)	48/50	98	DED Cataract	Thealoz Duo (TH 3% and HA 0.15%)	No tear substitutes or Hyabak (HA 0.15%)	No	No
Mencucci et al. [24] 2021	MN UM	1 ^b^	123 (83/40)	73.7 ± 9 (73.8 ± 9.2/73.6 ± 8.8)	77/46	123	DED Cataract	Thealoz Duo (TH 3% and HA 0.15%)	No tear substitutes	NR	No

Abbreviations: CB = Carbomer; CG = Control group; CoI = Conflict of interest; DM = Double-masked; DED = Dry eye disease; F = Female; F/U = Follow-up; HA = Acid hyaluronate; M = Male; MN = Monocentric; MT = Multicenter; NaCl = Sodium chloride; NR = Not reported; PG = Polyethylene glycol; RCTs = Randomized controlled trials; SM = Single-masked; TH = Trehalose; TG = Treatment group; UM = Unmasked. ^a^ Expressed as hours. ^b^ Expressed as months. ^c^ Expressed as mean ± SD or median [IQR], years.

**Table 2:** Baseline, Last visit and Differences (Last visit—Baseline) outcomes in the treatment group.

Author, Date	Assessment	OSDI (0–100)	TBUT, s	TFT, μm	TMH, mm	ST, mm	CFS (0–5)	VA^c^ (0–100)	Satisfaction
Schmidl et al. [12] 2015	Baseline	26.1 ± 5.1	5.4 ± 2.1	2.4 ± 0.4	NR	7.5 ± 7.1	NR	83.9 ± 8.2	NR
	Last visit	NR	6.3 ± 2.3	3.1 ± 0.9	NR	9.1 ± 9.7	NR	81.8 ± 18.3	
	Difference _LV-B_	-	0.9	0.7 ^a^	-	1.6	-	−2.1	
Wozniak et al. [11] 2016	Baseline	50.5 ± 20	3.45 ± 2.1	3.5 ± 0.7	NR	9.1 ± 5.5	NR	86.6 ± 4.7	High
	Last visit	NR	NR	3.8 ± 0.6	NR	NR	NR	NR	
	Difference _LV-B_	-	-	0.3	-	-	-	-	
Chiambaretta et al. [25] 2016	Baseline	45.4 ± 17.2	5.6 ± 1.9	NR	NR	8 ± 6.3	1.9 ± 1.5	NR	High
	Last visit	15.2 ± 18.7	8.3 ± 1.9	NR	NR	11 ± 6.2	0.5 ± 1.9	NR	
	Difference _LV-B_	−30.2 ^a^	2.7	-	-	3	−1.4 ^a^	-	
Doan et al. [26] 2018	Baseline	45.4 ± 17.2	NR	NR	NR	NR	NR	NR	NR
	Last visit	15.5 ± 13.9	NR	NR	NR	NR	NR	NR	
	Difference _LV-B_	−29.9 ^a^	-	-	-	-	-	-	
Caretti et al. [23] 2019	Baseline	31.2 ± 12.3	3.4 ± 1.1	NR	NR	NR	0.9 ± 0.6	66.4 ± 4.5	High
	Last visit	5.1 ± 7.9	6.6 ± 2.1	NR	NR	NR	0.00	84 ± 6.3	
	Difference _LV-B_	−26.1 ^a^	3.2 ^a^	-	-	-	−0.9 ^a^	17.6 ^a^	
Karaca et al. [27] 2020	Baseline	NR	NR	NR	2.3 ± 0.85	NR	NR	NR	High
	Last visit	NR	NR	NR	2.4 ± 0.76	NR	NR	NR	
	Difference _LV-B_	-	-	-	0.1	-	-	-	
Morya et al. [28] 2020	Baseline	NR	6.1 ± 3.4	NR	0.43 ± 0.2	6.1 ± 2.7	NR	NR	NR
	Last visit	NR	9.5 ± 3.3	NR	0.49 ± 0.3	11.4 ± 3.2	NR	NR	
	Difference _LV-B_	-	3.4 ^a^	-	0.06	5.3 ^a^	-	-	
Cagini et al. [13] 2021a	Baseline	8.2 ± 7.9	8 ± 2.5	NR	NR	11.8 ± 3	2.3 ± 0.5	NR	NR
	Last visit	1.2 ± 1.9	12.2 ± 3.2	NR	NR	14.6 ± 3.4	1.5 ± 0.5	NR	
	Difference _LV-B_	−7 ^a^	4.2 ^a^	-	-	2.8 ^a^	−0.8 ^a^	-	
Cagini et al. [15] 2021b	Baseline	44.5 ± 27.4	4.9 ± 2.2	NR	NR	7.1 ± 3.2	1.3 ± 0.8	71.2 ± 8.3	NR
	Last visit	7.8 ± 4.5	9.1 ± 1.3	NR	NR	9.5 ± 1.3	0.1 ± 0.4	87.5 ± 6.6	
	Difference _LV-B_	−36.7 ^a^	4.2 ^a^	-	-	2.4 ^a^	−1.2 ^a^	16.3	
Mencucci et al. [24] 2021	Baseline	20.4 ± 7.2	6.03 ± 1.7	NR	NR	10.5 ± 5.6	NR	NR	NR
	Last visit	15.8 ± 7.3	7.3 ±1.3	NR	NR	12.2 ± 8.7	NR	NR	
	Difference _LV-B_	−4.6 ^a^	1.3 ^a^	-	-	1.7	-	-	
	Mean ± SD ^b^	−22.4 ± 12.2	2.8 ± 1.2	0.5 ± 0.2	0.08 ± 0.1	2.8 ± 1.2	−1.1 ± 0.2	10.6 ± 8.9	

B = Baseline; CFS = Corneal fluorescein staining; LV = Last visit; NR = Not reported; OSDI = Ocular surface disease; SD = Standard deviation; ST = Schirmer test; TBUT = Tear break-up time; TFT = Tear film thickness; TMH = Tear meniscus height; VA = Visual acuity. ^a^ Statistical significance level *p* < 0.05. ^b^ Mean ± SD values of the difference _LV-B_ for each variable. ^c^ Early treatment diabetic retinopathy study (ETDRS) letter score.

**Table 3:** Baseline, Last visit and Differences (Last visit—Baseline) outcomes in the control group.

Author, Date	Assessment	OSDI (0–100)	TBUT, s	TFT, μm	TMH, mm	ST, mm	CFS (0–5)	VA ^c^ (0–100)	Satisfaction
Schmidl et al. [12] 2015	Baseline	27.1 ± 4.8	4.9 ± 2.1	2.5 ± 0.4	NR	12.1 ± 9.5	NR	84.7 ± 3.2	NR
	Last visit	NR	5.4 ± 2.8	2.9 ± 0.5	NR	14.8 ± 10	NR	85.1 ± 18.3	
	Difference _LV-B_	-	0.5	0.4 ^a^	-	2.7	-	0.4	
Wozniak et al. [11] 2016	Baseline	50.4 ± 17.9	3.6 ± 1.7	3.5 ± 0.7	NR	9.9 ± 8	NR	87.1 ± 3.6	High
	Last visit	NR	NR	3.6 ± 0.05	NR	NR	NR	NR	
	Difference _LV-B_	-	-	0.1	-	-	-	-	
Chiambaretta et al. [25] 2016	Baseline	46 ± 18.7	4.9 ± 1.5	NR	NR	6.8 ± 4.7	2.1 ± 1.6	NR	High
	Last visit	20.4 ± 16.4	7.5 ± 1.2	NR	NR	10.5 ± 6.1	1.3 ± 1.5	NR	
	Difference _LV-B_	−25.6 ^a^	2.6	-	-	3.7	−0.8 ^a^	-	
Doan et al. [26] 2018	Baseline	46 ± 18.7	NR	NR	NR	NR	NR	NR	NR
	Last visit	17.8 ± 13.9	NR	NR	NR	NR	NR	NR	
	Difference _LV-B_	−28.2 ^a^	-	-	-	-	-	-	
Caretti et al. [23] 2019	Baseline	21 ± 12.7	4.1 ± 1.5	NR	NR	NR	0.65 ± 0.5	68.3 ± 3.8	High
	Last visit	11.2 ± 11.4	5.1 ± 1.4	NR	NR	NR	0.28 ± 0.4	84.6 ± 6.5	
	Difference _LV-B_	−9.8 ^a^	1	-	-	-	−0.3	16.3 ^a^	
Karaca et al. [27] 2020	Baseline	NR	NR	NR	2.4 ± 0.7	NR	NR	NR	High
	Last visit	NR	NR	NR	2,4 ± 0.4	NR	NR	NR	
	Difference _LV-B_	-	-	-	0.1	-	-	-	
Morya et al. [28] 2020	Baseline	NR	5.4 ± 3.4	NR	0.45 ± 0.2	6.1 ± 2.7	NR	NR	NR
	Last visit	NR	7.7 ± 3.3	NR	0.47 ± 0.3	9.8 ± 3.2	NR	NR	
	Difference _LV-B_	-	2.3 ^a^	-	0.02	3.7 ^a^	-	-	
Cagini et al. [13] 2021a	Baseline	9.3 ± 9.4	10 ± 3.8	NR	NR	13.1 ± 4.4	2.2 ± 0.5	NR	NR
	Last visit	4.8 ± 3.4	9.7 ± 3.5	NR	NR	12.6 ± 4	1.9 ± 0.6	NR	
	Difference _LV-B_	−4.5 ^a^	−0.3 ^a^	-	-	−0.5	−0.2 ^a^	-	
Cagini et al. [15] 2021b	Baseline	37.1 ± 19.8	5.2 ± 2.2	NR	NR	7.2 ± 3.1	1.1 ± 0.7	73.2 ± 8.1	NR
	Last visit	20.4 ± 13.1	6.1 ± 1.3	NR	NR	8.4 ± 1.5	0.7 ± 0.4	87.2 ± 5.8	
	Difference _LV-B_	−16.7 ^a^	0.9 ^a^	-	-	1.2 ^a^	−0.4 ^a^	14	
Mencucci et al. [24] 2021	Baseline	21.1 ± 6.7	6.7 ± 1.7	NR	NR	11.7 ± 6.4	NR	NR	NR
	Last visit	22.3 ± 6.4	6.48 ± 1.4	NR	NR	12.4 ± 8.3	NR	NR	
	Difference _LV-B_	1.2	−0.22	-	-	0.7	-	-	
	Mean ± SD ^b^	−14.3 ± 10.1	0.9 ± 1	0.25 ± 0.2	0.05 ± 0.1	1.9 ± 1.6	−0.4 ± 0.2	10.2 ± 7	

B = Baseline; CFS = Corneal fluorescein staining; LV = Last visit; NR = Not reported; OSDI = Ocular surface disease; SD = Standard deviation; ST = Schirmer test; TBUT = Tear break-up time; TFT = Tear film thickness; TMH = Tear meniscus height; VA = Visual acuity. ^a^ Statistical significance level *p* < 0.05. ^b^ Mean ± SD values of the difference _LV-B_ for each variable. ^c^ Early treatment diabetic retinopathy study (ETDRS) letter score.

**Table 4:** Inter-group differences [(T group _LV-B_)—(C group _LV-B_)] as to outcomes.

Author, Date	Assessment	OSDI (0–100)	TBUT, s	TFT, μm	TMH, mm	ST, mm	CFS (0–5)	VA ^b^ (0–100)	F/A
Schmidl et al. [12] 2015	T difference _LV-B_	-	0.9	0.7 ^a^	-	1.6	-	−2.1	F
	C difference _LV-B_	-	0.5	0.4 ^a^	-	2.7	-	0.4	
	Difference _T-C_	-	0.4	0.3 ^a^	-	−1.1	-	−2.5	
Wozniak et al. [11] 2016	T difference _LV-B_	-	-	0.3	-	-	-	-	F
	C difference _LV-B_	-	-	0.1	-	-	-	-	
	Difference _T-C_	-	-	0.2	-	-	-	-	
Chiambaretta et al. [25] 2016	T difference _LV-B_	−30.2 ^a^	2.7	-	-	3	−1.4 ^a^	-	F
	C difference _LV-B_	−25.6 ^a^	2.6	-	-	3.7	−0.8 ^a^	-	
	Difference _T-C_	−4.6	0.1	-	-	−0.7	−0.6	-	
Doan et al. [26] 2018	T difference _LV-B_	−29.9 ^a^	-	-	-	-	-	-	F
	C difference _LV-B_	−28.2 ^a^	-	-	-	-	-	-	
	Difference _T-C_	−1.7	-	-	-	-	-	-	
Caretti et al. [23] 2019	T difference _LV-B_	−26.1 ^a^	3.2 ^a^	-	-	-	−0.9 ^a^	17.6 ^a^	F
	C difference _LV-B_	−9.8 ^a^	1	-	-	-	−0.3	16.3 ^a^	
	Difference _T-C_	−16.3	2.2	-	-	-	−0.6	1.3	
Karaca et al. [27] 2020	T difference _LV-B_	-	-	-	0.1	-	-	-	F
	C difference _LV-B_	-	-	-	0.1	-	-	-	
	Difference _T-C_	-	-	-	0.00	-	-	-	
Morya et al. [28] 2020	T difference _LV-B_	-	3.4 ^a^	-	0.06	5.3 ^a^	-	-	F
	C difference _LV-B_	-	2.3 ^a^	-	0.02	3.7 ^a^	-	-	
	Difference _T-C_	-	1.1	-	0.04	1.6	-	-	
Cagini et al. [13] 2021a	T difference _LV-B_	−7 ^a^	4.2 ^a^	-	-	2.8 ^a^	−0.8 ^a^	-	F
	C difference _LV-B_	−4.5 ^a^	−0.3 ^a^	-	-	−0.5	−0.2 ^a^	-	
	Difference _T-C_	−2.5	4.5	-	-	3.3	−0.6	-	
Cagini et al. [15] 2021b	T difference _LV-B_	−36.7 ^a^	4.2 ^a^	-	-	2.4 ^a^	−1.2 ^a^	16.3	F
	C difference _LV-B_	−16.7 ^a^	0.9 ^a^	-	-	1.2 ^a^	−0.4 ^a^	14	
	Difference _T-C_	−20	3.3	-	-	1.2 ^a^	−0.8 ^a^	2.3	
Mencucci et al. [24] 2021	T difference _LV-B_	−4.6 ^a^	1.3 ^a^	-	-	1.7	-	-	F
	C difference _LV-B_	1.2	−0.2	-	-	0.7	-	-	
	Difference _T-C_	−5.8	1.5	-	-	1	-	-	

B = Baseline; CFS = Corneal fluorescein staining; F/A = Favor/against; LV = Last visit; NR = Not reported; OSDI = Ocular surface disease; SD = Standard deviation; ST = Schirmer test; TBUT = Tear break-up time; TFT = Tear film thickness; TMH = Tear meniscus height; VA = Visual acuity. ^a^ Statistical significance level *p* < 0.05. ^b^ Early treatment diabetic retinopathy study (ETDRS) letter score.

## Data Availability

Not applicable.
